# In Situ Gelling of TPZ-Loaded Nanogel for Sustained Intratumoral Delivery and Enhanced Cancer Immunotherapy

**DOI:** 10.3390/gels12090781

**Published:** 2026-09-01

**Authors:** Ling Li, Kexin Wang, Zhe Song, Hongan Tian, Cai Wang, Ling Zhang, Houqiang Yu

**Affiliations:** 1 School of Biomedical Engineering, Xianning Medical College, Hubei University of Science and Technology, Xianning 437100, China; 2 School of Pharmacy, Xianning Medical College, Hubei University of Science and Technology, Xianning 437100, China; 3 Xianning Medical College, Hubei University of Science and Technology, Xianning 437100, China; 4 Department of Mathematics and Statistics, Hubei University of Science and Technology, Xianning 437100, China

**Keywords:** TPZ, breast cancer, thermosensitive nanogel, intratumoral injection

## Abstract

TPZ is a hypoxia-activated anticancer agent that exerts selective cytotoxicity within hypoxic tumor microenvironments. Although intratumoral injection of TPZ can achieve localized antitumor activity, the drug is prone to rapid diffusion and clearance from the injection site, limiting its long-term retention and thereby reducing therapeutic durability and overall efficacy. To achieve sustained release and prolonged retention of TPZ in subcutaneous tumors, a thermosensitive nanogel loaded with TPZ (TPZ@PNA-TNG) was developed. By thoroughly mixing 3 mg/mL TPZ with 6 wt% PNA-TNG, the resulting formulation enabled continuous slow release of TPZ following intratumoral injection, resulting in favorable therapeutic outcomes. The sol–gel phase transition behavior of TPZ@PNA-TNG was investigated using the inverted vial method and rheological measurements. In vivo antitumor studies demonstrated that a single administration of TPZ@PNA-TNG effectively suppressed tumor progression, with tumor volume decreasing to 0.72 ± 0.04 times its initial size over a 14-day period. Mechanistically, TPZ@PNA-TNG markedly enhanced antitumor immune responses, inhibited tumor cell proliferation, promoted apoptosis and anti-angiogenesis, and ultimately induced extensive ischemic necrosis within subcutaneous tumors. In addition, owing to the prolonged intratumoral retention capability of PNA-TNG, TPZ@PNA-TNG enabled sustained local delivery of TPZ, thereby significantly reducing systemic toxicity and adverse effects associated with TPZ while maintaining favorable biocompatibility. These findings highlight the therapeutic potential of TPZ@PNA-TNG for cancer therapy applications.

## 1. Introduction

Globally, breast cancer is the most commonly detected malignancy and the leading cause of cancer-related deaths among women, representing about 25% of new cancer cases and 16% of cancer deaths [1,2,3,4]. The clinical management of this disease is significantly challenged by its highly invasive and metastatic behavior. Despite the proven utility of traditional approaches, such as surgical excision, chemotherapy, radiation therapy, immunotherapy, and molecular targeted therapy [5,6,7], monotherapy regimens rarely result in a complete cure and are frequently accompanied by considerable adverse effects. Given these limitations, the exploration of multimodal treatment strategies has become a central focus in advancing therapeutic success [8,9].

TPZ, a hypoxia-responsive prodrug, is preferentially activated inside the hypoxic regions of tumors, which arise from rapid proliferation and aberrant vasculature [10,11,12]. Upon activation, it induces single- and double-strand DNA breaks, base damage, and subsequent cell death, thereby selectively targeting hypoxic cells in solid tumors [13]. TPZ requires a hypoxic environment for activation to exert its antitumor effects. Therefore, it is commonly used in combination with TACE, where embolization of tumor-feeding vessels induces a localized hypoxic microenvironment, thereby promoting the selective activation of TPZ. However, this reliance on artificially induced hypoxia also imposes certain limitations on its clinical application, particularly in terms of therapeutic scope and consistency of efficacy [14,15]. Alternative strategies, such as combination with vascular-disrupting agents, have also been explored to enhance TPZ activation within tumors [16]. Intratumoral injection facilitates direct drug delivery to the tumor site, achieving high local concentrations and a more immediate therapeutic impact on cancer cells [17]. Accordingly, intratumoral injection represents a promising strategy to overcome the limitations of systemic delivery and hypoxia-dependent activation of TPZ, enabling more controlled and localized therapeutic effects. To further enhance this localized strategy, thermosensitive nanogels have been developed as carriers to improve drug retention and release at the tumor site. To highlight the advantages of our TPZ@PNA‑TNG over previously reported TPZ delivery systems, various strategies—including hypoxia‑responsive core‑shell polymeric nanoparticles, ROS‑responsive dendrimer nanogels, and thermosensitive hydrogels—have been explored to improve TPZ delivery. Although these systems have advanced TPZ delivery and hypoxic cytotoxicity, they still have limitations: hypoxia‑responsive core‑shell polymeric nanoparticles are largely dependent on systemic circulation for tumor accumulation [18], dendrimer‑based nanogels are mostly designed for systemic intravenous administration and are easily cleared in vivo [19], and conventional injectable thermosensitive hydrogels still face challenges in optimizing local tumor retention capacity [20]. In contrast, our TPZ@PNA-TNG offers minimally invasive intratumoral injection and sol–gel transition at physiological temperature to form an in situ depot, which prolongs drug retention, enhances efficacy, and reduces systemic exposure. Based on these considerations, we investigated its therapeutic efficacy and biosafety in the 4T1 subcutaneous tumor model, a widely used murine breast cancer model that recapitulates tumor growth and metastasis [21,22], focusing on whether this combination could improve TPZ accumulation in hypoxic regions, ultimately aiming to provide a more effective strategy for breast cancer treatment.

**Scheme 1:**
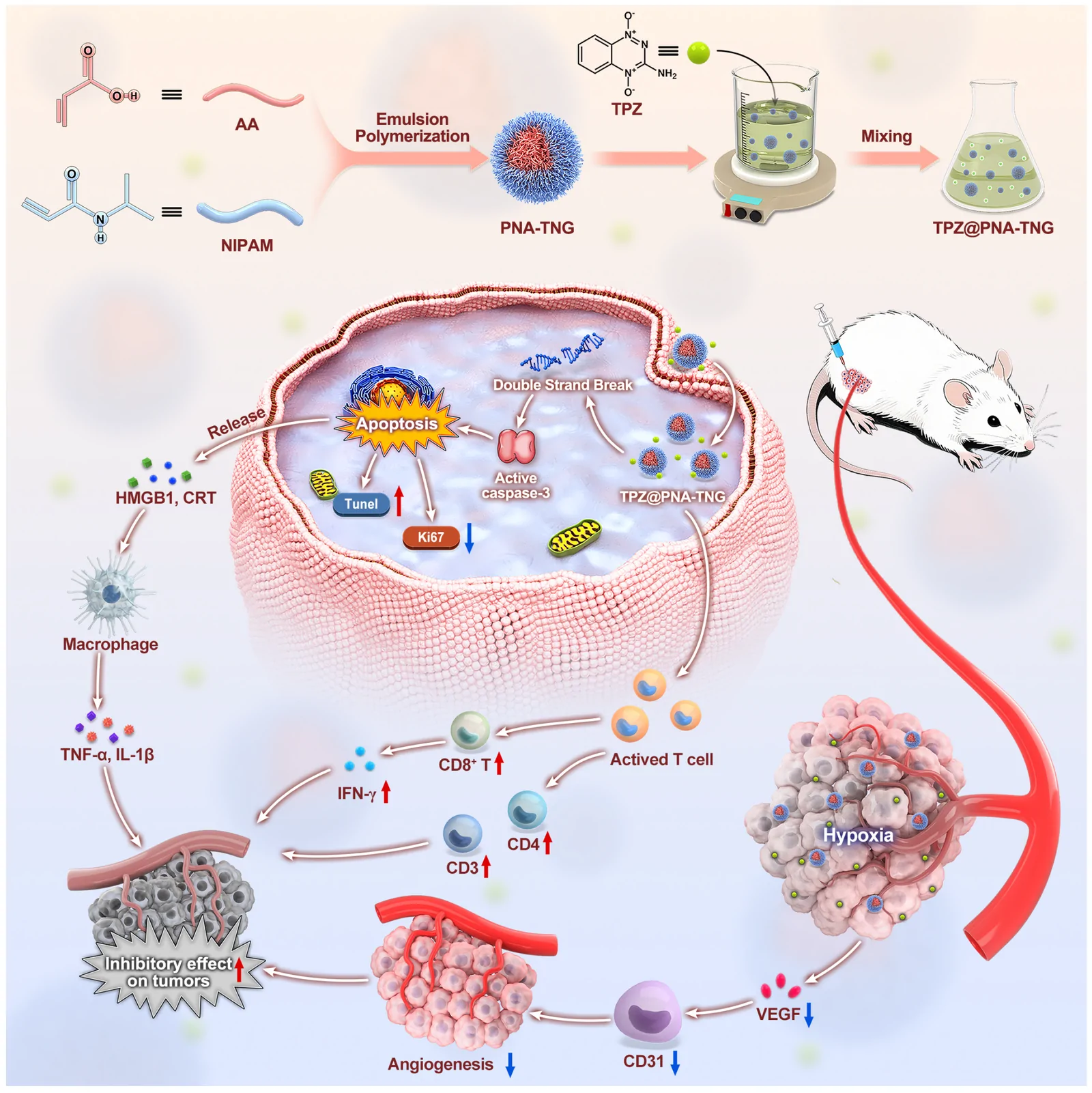
Schematic illustration of TPZ temperature-sensitive nano-gel preparation and its tumor treatment efficacy. Red arrows indicate an increase; Blue arrows indicate a decrease.

PNA-TNG thermosensitive nanogels represent a novel drug delivery system with considerable advantages for tumor therapy [23,24]. In their liquid state at room temperature, they allow for convenient injection. Upon intratumoral administration, they undergo a sol–gel transition triggered by the slightly elevated temperature of tumor tissue relative to normal physiological temperature [25,26,27,28]. This hydrophilic-to-hydrophobic transition facilitates their localization and long-term retention at the tumor site. Beyond their role as drug delivery carriers, nanogel platforms have also demonstrated immunomodulatory functions that may contribute to antitumor therapy [29]. Moreover, when loaded with TPZ, the nanogel system not only increases local drug concentration within the tumor but also reduces systemic exposure and associated side effects on healthy tissues (Scheme 1).

## 2. Results and Discussion

### 2.1. Characterization of PNA-TNG

**Figure 1:**
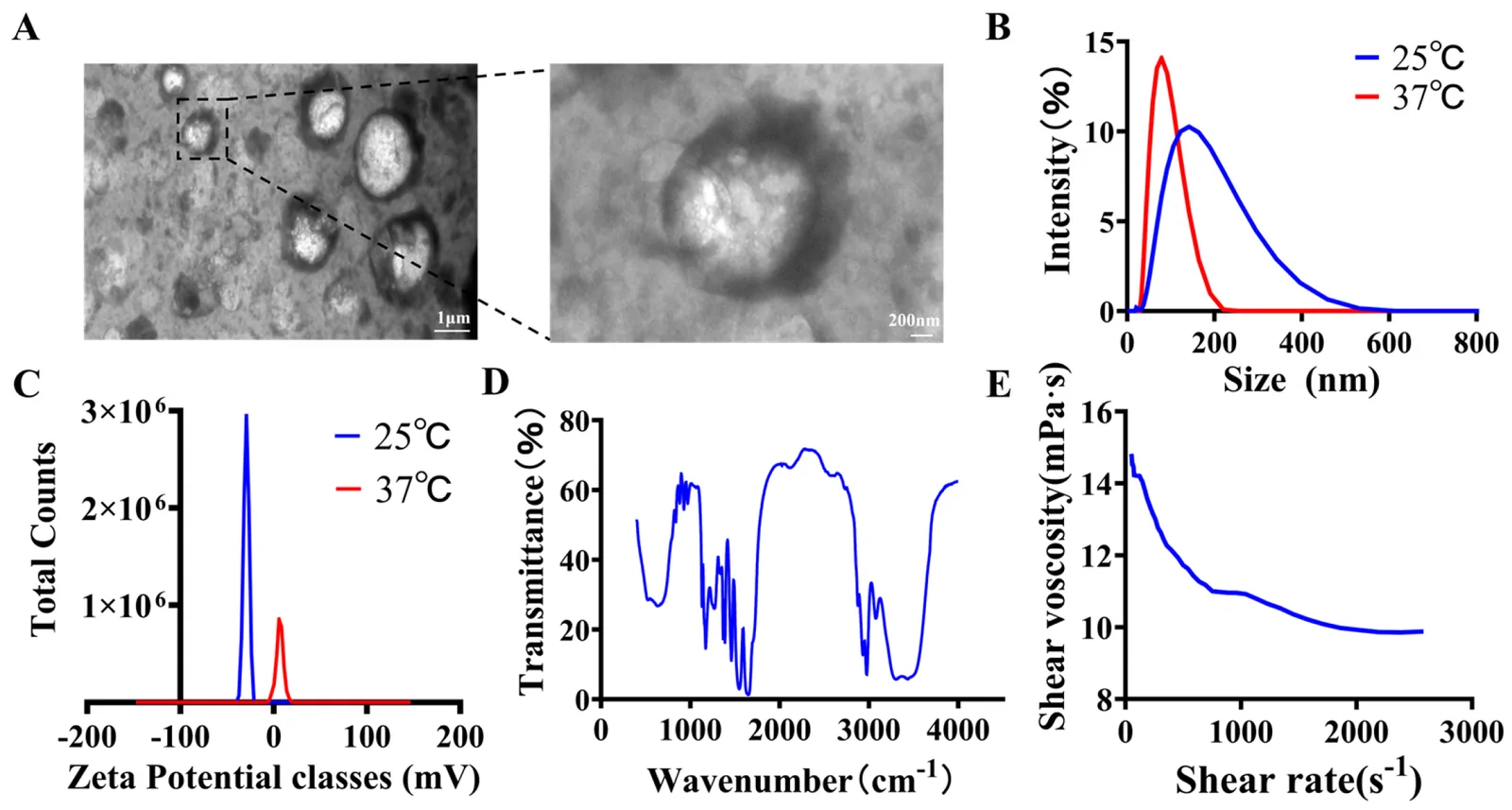
Physicochemical characterization of PNA-TNG. (**A**) TEM images. Scale bars: 1 µm, 200 nm. (**B**) DLS analysis. (**C**) Zeta-potential measurement. (**D**) FTIR analysis. (**E**) Shear viscosity under different shear rates.

TEM images show uniform dispersion of PNA-TNG (Figure 1A) and reveal a distinct core–shell structure. DLS analysis revealed average hydrodynamic diameters of 141.77 ± 4.8 nm (PDI = 0.23 ± 0.09) at 25 °C and 78.82 ± 3.1 nm (PDI = 0.19 ± 0.05) at 37 °C, with the smaller size at physiological temperature confirming the prominent thermosensitivity of PNA-TNG (Figure 1B). The zeta potential of PNA-TNG shifted from −29.12 mV at 25 °C to 5.84 mV at 37 °C (Figure 1C), indicating a marked temperature-dependent surface charge variation.

The chemical structure of PNA-TNG was characterized by FTIR spectroscopy, which revealed characteristic absorption bands corresponding to the functional groups of the constituent monomers. The amide I band at 1638 cm^−1^ was attributed to C=O stretching vibrations, while the amide II band at 1542 cm^−1^ corresponded to N–H bending vibrations coupled with C–N stretching. In addition, a distinct peak at 1690 cm^−1^ was assigned to the carboxyl groups from acrylic acid. These spectral features collectively demonstrated the successful incorporation of both amide and carboxyl functionalities into the PNA-TNG network (Figure 1D). Rheological measurements showed that the viscosity of PNA-TNG decreased from 14.7 mPa·s at a shear rate of 53 s^−1^ to 9.8 mPa·s at 2548 s^−1^. This shear-thinning behavior indicates improved flowability under applied shear stress, consistent with the characteristic rheological properties of thermosensitive nanogels (Figure 1E).

The 2 wt% and 4 wt% gels showed no phase transition with temperature variations, whereas the 8 wt% hydrogel underwent a sol–gel transition at 35 °C but exhibited instability upon further heating. The 6 wt% dispersion remained stable up to 40 °C, above which partial water precipitation was observed (Figure S1). The temperature-responsive behavior of PNA-TNG nanogels was further evaluated by observing their morphological states at 25 °C and 37 °C. At room temperature (25 °C), the nanogels were uniformly dispersed in the liquid phase, existing in a sol state with good fluidity and no notable morphological changes. Upon heating to 37 °C, the nanogels underwent a distinct sol–gel phase transition, confirming their excellent thermosensitive performance (Figure S2, Vidios S1 and S2).

### 2.2. Characterization of TPZ@PNA-TNG

To determine the optimal TPZ loading concentration, TPZ at 1, 3, 5, and 7 mg/mL was incorporated into 6% PNA-TNG dispersions and homogenized for 30 min in the dark. The sol–gel transition was evaluated by the tube-inversion method. The 7 mg/mL group required 40 °C to complete gelation, incompatible with the tumor microenvironment, and was thus excluded (Figure S3). Stability assessment showed that the 1 and 5 mg/mL groups exhibited phase separation within 10 days at room temperature, whereas the 3 mg/mL group maintained favorable homogeneity and structural stability throughout the observation period (Figure S4). Microscopic imaging further verified that the 1 and 5 mg/mL formulations had uneven particle distribution and irregular microstructure, while 3 mg/mL TPZ@PNA-TNG displayed uniform dispersion and regular morphology (Figure S5). Accordingly, 3 mg/mL was selected as the optimal concentration for subsequent experiments.

**Figure 2:**
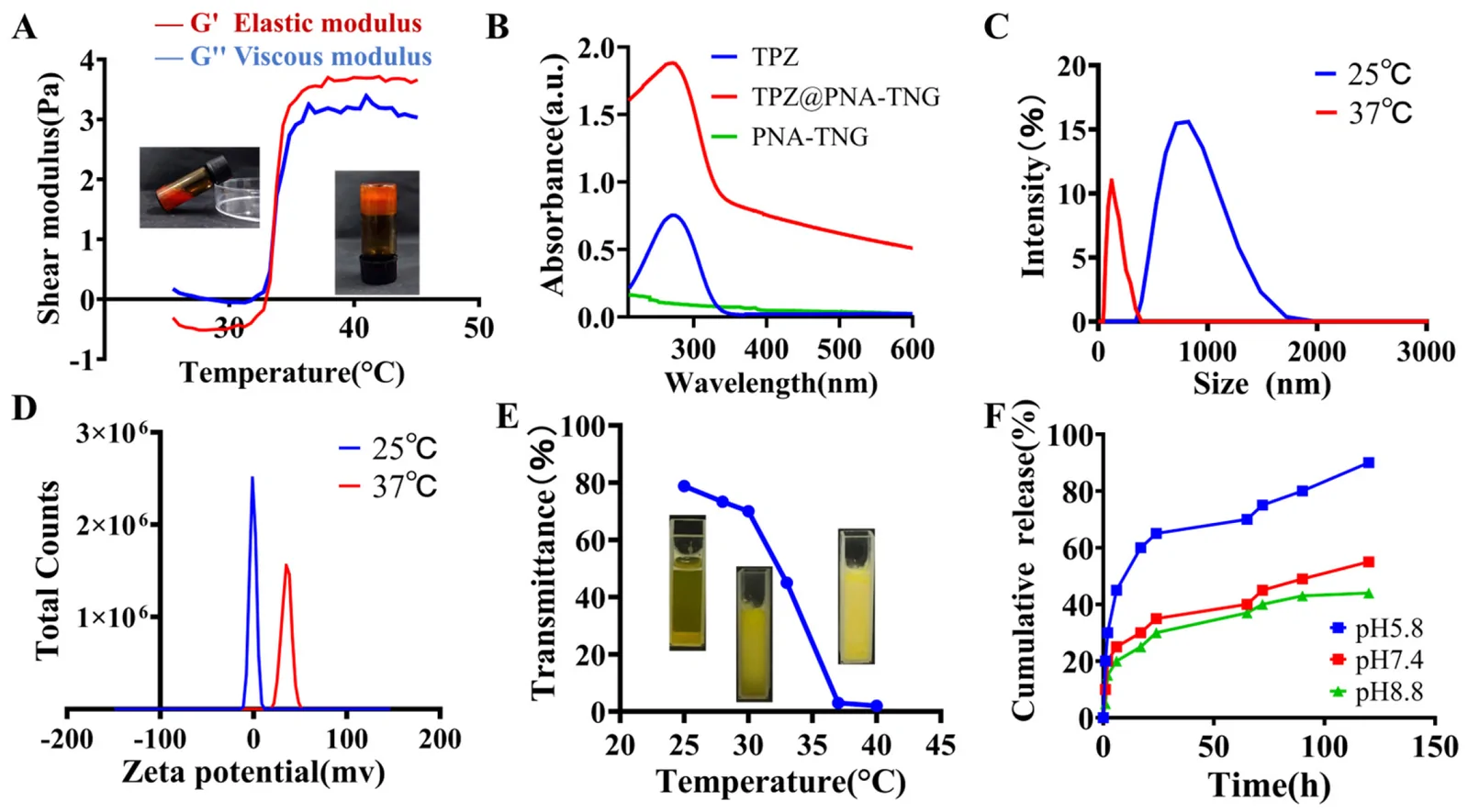
Characterization of TPZ@PNA-TNG nanogels. (**A**) Rheological temperature sweep curves. (**B**) UV–Vis absorption spectrum. (**C**) Particle size variation with temperature. (**D**) Zeta potential change with temperature. (**E**) Transmittance curve at different temperatures. (**F**) Drug release profiles at different pH values.

The EE% and DL% of TPZ@PNA-TNG were determined to be approximately 66% and 11%, respectively, indicating efficient drug incorporation (Figure S6). Three independent batches were prepared under identical conditions, and the particle size, PDI, zeta potential, EE%, and DL% were measured for each. The relative standard deviations (RSDs) for all parameters were below 5%, confirming good batch-to-batch reproducibility. Rheological measurementd further validated the thermoresponsive sol–gel transition: both G′ and G″ slightly decreased at 25–27 °C, then increased sharply with further heating; the crossover point marked the transition temperature, confirming successful gelation (Figure 2A). UV–Vis full-wavelength scanning (200–600 nm) showed that the maximum absorption wavelength of TPZ@PNA-TNG was at 270 nm, which served as the detection wavelength for subsequent quantitative analysis (Figure 2B).

The temperature-dependent particle size variation further reflected the thermosensitive structural evolution of TPZ@PNA-TNG. At 25 °C, the average particle size of TPZ@PNA-TNG was 825.46 ± 12.23 nm (PDI = 0.21 ± 0.03), consistent with the microscopic morphology observations. Upon heating to 37 °C, enhanced hydrophobic interactions induced molecular chain contraction, leading to a significant decrease in particle size to 131.96 ± 8.4 nm (PDI = 0.19 ± 0.05), at which equilibrium was reached (Figure 2C). Meanwhile, the zeta potential shifted from −1.6 mV at 25 °C to 35.5 mV at 37 °C (Figure 2D). Temperature-dependent transmittance measurements at 450 nm revealed that, over the range of 25–40 °C, the transmittance of TPZ@PNA-TNG gradually decreased with increasing temperature (Figure 2E). This reduction in transmittance further confirmed the temperature-triggered hydrophilic-to-hydrophobic transition and structural shrinkage of the nanogel network.

The pH-responsive drug release behavior of TPZ@PNA-TNG was evaluated under physiological (pH 7.4), weakly alkaline (pH 8.8), and tumor acidic (pH 5.8) conditions. At pH 7.4 and pH 8.8, the release of TPZ was relatively slow and sustained, with the lowest cumulative release observed at pH 8.8. In contrast, the acidic environment at pH 5.8 significantly accelerated the initial drug release and led to a higher cumulative release within 30 h. This pH-dependent release profile is consistent with the characteristic tumor microenvironment, where anaerobic glycolysis results in lactic acid accumulation and local acidification. Such acid-responsive release behavior may enable TPZ@PNA-TNG to achieve targeted and rapid drug release at tumor sites, thereby supporting effective therapy against 4T1 tumors (Figure 2F).

### 2.3. TPZ@PNA-TNG Exhibits Potent Therapeutic Efficacy Against 4T1 Subcutaneous Tumors

**Figure 3:**
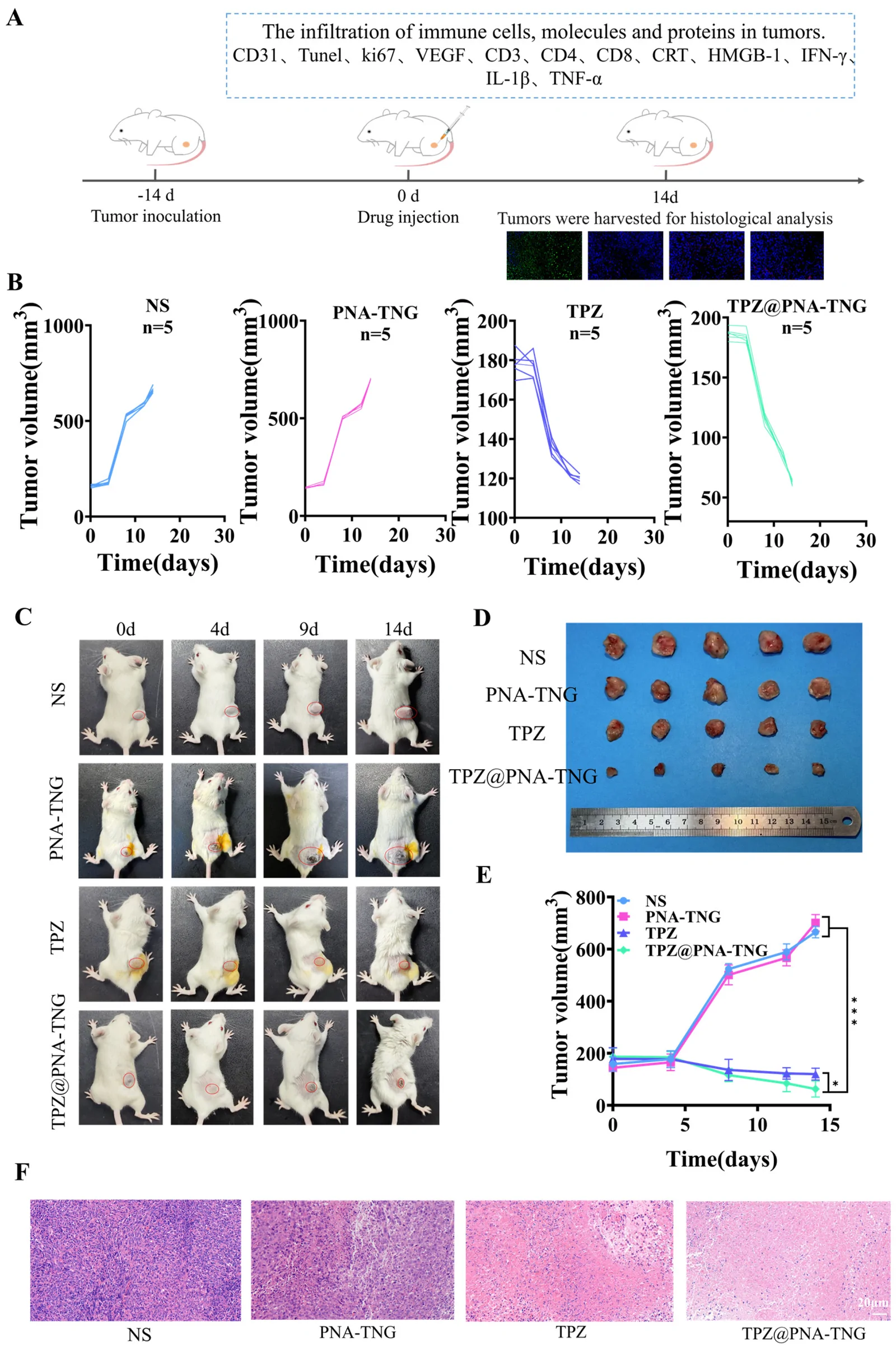
In vivo antitumor efficacy. (**A**) Schematic illustration of the in vivo experimental schedule. (**B**) Individual tumor growth curves of mice in four groups (NS, PNA-TNG, TPZ, and TPZ@PNA-TNG; n = 5 per group). (**C**) Representative photographs of tumor-bearing mice at different time points. Red circles indicate tumors. (**D**) Excised tumor tissues on day 14. (**E**) Average tumor volumes over time. All data are presented as mean ± SD (n = 5). Statistical significance was determined by one-way ANOVA followed by Dunnett’s post-hoc test, with comparisons made against the TPZ@PNA-TNG group. * *p* < 0.05, *** *p* < 0.001. (**F**) H&E staining of tumor sections after 14 days.

In a well-established subcutaneous tumor model (Figure 3A), longitudinal measurements of tumor size revealed that TPZ@PNA-TNG was the most effective inhibitor of 4T1 tumor growth. The TPZ monotherapy group exhibited a discernible but moderate effect, while the NS and PNA-TNG controls exhibited no inhibition (Figure 3B). This trend was visually confirmed by representative images of the mice, where the tumor areas (circled in red) expanded in the control groups but were markedly contained and reduced in the TPZ@PNA-TNG group over the 14-day period (Figure 3C). Consistent with these observations, the analysis of excised tumors at the study endpoint confirmed that the TPZ@PNA-TNG group yielded the smallest tumor volumes among all groups (Figure 3D).

Tumor growth progressed rapidly in the NS and PNA-TNG control groups. Although TPZ monotherapy resulted in a modest retardation of tumor growth, the TPZ@PNA-TNG treatment induced a marked regression in tumor volume (Figure 3E). Histological assessment by H&E staining further corroborated these findings. Tumor tissues from the NS group displayed densely packed cells with well-preserved nuclear morphology and high proliferative activity, with no significant necrosis or apoptosis. The PNA-TNG group presented with irregular cell morphology and maintained high cellular density, similarly lacking appreciable apoptosis, which indicates a negligible inhibitory effect. In contrast, the TPZ group exhibited increased extracellular matrix, sparse nuclear distribution, and visible cell death, confirming its cytotoxic potential. Most notably, the TPZ@PNA-TNG group demonstrated advanced nuclear pyknosis and necrosis, with a further reduced and dispersed cell population, underscoring the superior inhibitory and cytotoxic efficacy of the combined therapy (Figure 3F).

### 2.4. Tumor Microenvironment Evaluation

**Figure 4:**
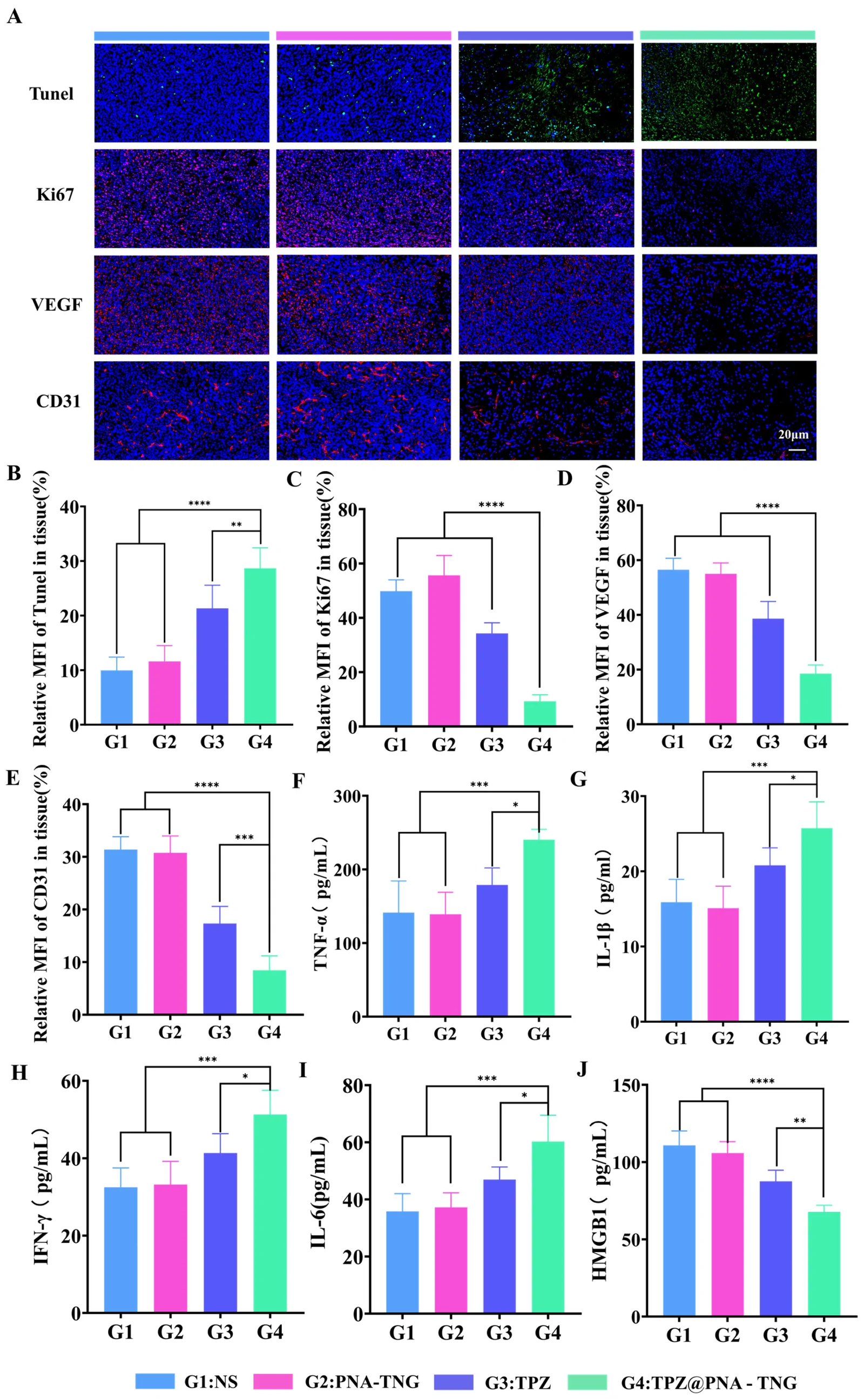
Immunofluorescence staining and cytokine expression analysis of tumor tissues. (**A**) Representative immunofluorescence images of tumor sections stained for TUNEL, Ki67, VEGF, and CD31 (scale bar = 20 μm). (**B**–**E**) Quantitative analysis of relative mean fluorescence intensity (MFI) in tumor tissues for TUNEL, Ki67, VEGF, and CD31, respectively. (**F**–**J**) Concentrations of inflammation-associated and immune-regulatory cytokines (TNF-α, IL-1β, IFN-γ, IL-6, and HMGB1) measured in tumor homogenates collected on day 14 post-administration. All data are presented as mean ± SD (n = 5). Statistical significance was determined by one-way ANOVA followed by Dunnett’s post-hoc test, with comparisons made against the TPZ@PNA-TNG group. * *p* < 0.05, ** *p* < 0.01, *** *p* < 0.001, **** *p* < 0.0001.

TUNEL [30] staining reflects tumor cell apoptosis by labeling apoptotic cells. In the TPZ@PNA-TNG group, TUNEL-stained nuclei exhibited higher green fluorescence signals, indicating significant apoptosis. Fewer green-marked cells were observed in the NS and PNA-TNG groups, suggesting minimal inhibitory effects on 4T1 cells. Ki67 [31,32] staining, which labels proliferating cells to assess tumor cell proliferation, showed significantly fewer proliferative cells in TPZ@PNA-TNG compared to other groups, indicating effective suppression of cancer cell growth. VEGF [33,34], a marker of vascular endothelial growth factor, was evaluated for tumor angiogenesis. The TPZ@PNA-TNG group demonstrated significantly lower VEGF expression than the other three groups, further confirming its most notable anti-tumor effect. CD31 [35] staining, which labels vascular endothelial cells to reflect tumor vascular density, revealed fewer red fluorescence signals in TPZ@PNA-TNG, demonstrating effective inhibition of tumor angiogenesis (Figure 4A). The results were further confirmed by quantitative fluorescence analysis (Figure 4B–E).

CD3 is a core marker expressed on the surface of T lymphocytes, and its expression level is associated with the status of cellular immune function [36,37]. Immunohistochemical analysis showed significantly higher numbers of CD3^+^ cells in tumor tissues from the TPZ@PNA-TNG treatment group compared to the NS and PNA-TNG control groups, suggesting increased infiltration of activated T cells and a potential activation of antitumor cellular immunity (Figure S7). CD8^+^ T cells play an important role in tumor elimination, and their abundance and activity are closely associated with immunotherapeutic efficacy [38]. Immunohistochemical analysis demonstrated that tumor tissues from the TPZ@PNA-TNG treatment group exhibited significantly higher CD8^+^ T-cell infiltration than those from the NS and PNA-TNG control groups, which may contribute to enhanced direct tumor cell killing and improved therapeutic outcomes (Figure S8). CRT [39], a calcium-binding protein primarily located in the endoplasmic reticulum of eukaryotic cells, translocates to the tumor cell surface upon chemotherapeutic stimulation. This translocation allows CRT to serve as a signal molecule for immune recognition. The TPZ@PNA-TNG complex releases TPZ in a temperature-responsive manner. Upon activation under hypoxic conditions, TPZ induces tumor cell death, which is accompanied by the surface exposure of CRT. This process may facilitate the uptake and processing of tumor antigens by DCs, thereby contributing to the initiation of an antitumor immune response (Figure S9).

Serum levels of TNF-α in serum after various treatments were measured using ELISA. The serum TNF-α levels [40] in the TPZ group and TPZ@PNA-TNG group were significantly higher than those in the NS group and PNA-TNG group. The elevated TNF-α levels in the TPZ group suggested that TPZ may induce apoptosis in tumor cells and promote the release of related inflammatory factors. In the TPZ@PNA-TNG group, the marked increase in TNF-α levels was consistent with enhanced immune activation, suggesting that the nanogel formulation may facilitate TPZ-mediated TNF-α secretion and thereby contribute to the regulation of the tumor immune microenvironment (Figure 4F). Furthermore, IL-1β levels [41] in tumor tissues and mouse serum from the TPZ@PNA-TNG treatment group were significantly higher than those from the NS and PNA-TNG groups. These findings suggest that the treatment may not only eliminate tumor cells but also stimulate immune cells to produce IL-1β in response to tumor cell debris, potentially initiating an inflammatory-immune cascade (Figure 4G and Figure S10). CD4^+^ T cells [42] play a key role in modulating the intensity and direction of immune responses. Experimental results showed that tumor tissues in the TPZ@PNA-TNG treatment group exhibited significantly increased CD4^+^ T-cell infiltration, suggesting that this therapy may activate regulatory T cells and steer immune responses toward an antitumor phenotype (Figure S11).

During this process, the cytokine IFN-γ [43,44] secreted by CD4^+^ T cells may not only enhance the cytotoxicity of CD8^+^ T cells but also inhibit tumor angiogenesis. These findings suggest that the antitumor efficacy of TPZ@PNA-TNG may be partially mediated through CD4^+^ T-cell-dependent immune responses (Figure 4H and Figure S12). IL-6 [45,46] is a pivotal molecule in the inflammatory response and immune regulation, functioning through binding to IL-6 receptors on target cell surfaces. The elevated IL-6 levels in tumor tissues or serum from the TPZ@PNA-TNG treatment group suggest that IL-6 may exert pro-inflammatory and immune-activating effects, potentially involving T-cell differentiation, NK cell activation, and enhanced CD8^+^ T-cell cytotoxicity, thereby contributing to the antitumor immune response (Figure 4I). HMGB1 [47], a high-mobility group box 1 protein, is a non-histone chromatin protein found in the nucleus of eukaryotic cells. Upon cell damage or necrosis, HMGB1 is released from the nucleus into the extracellular space. During tumor cell apoptosis, HMGB1 may also be released through secondary necrosis. In the TPZ@PNA-TNG treatment group, the release of HMGB1 was significantly increased, which may enhance its cytotoxic efficacy against tumor cells (Figure 4J and Figure S13).

### 2.5. Biocompatibility Evaluation

**Figure 5:**
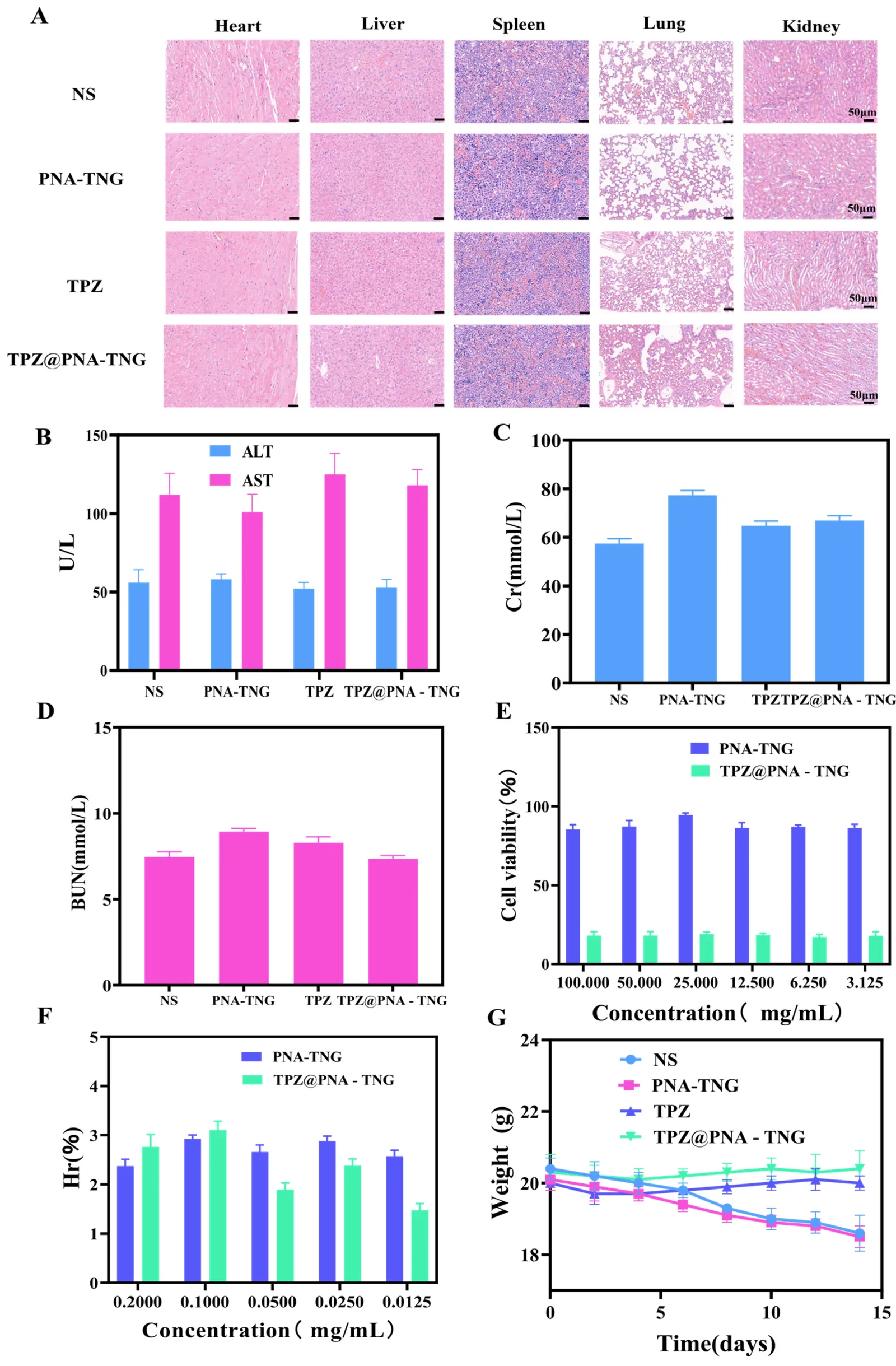
Biocompatibility evaluation. (**A**) H&E staining of heart, liver, spleen, lung, and kidney after various treatments (scale bar = 50 μm). (**B**–**D**) Serum levels of ALT, AST, Cr, and BUN in each group. (**E**) Cytotoxicity of 4T1 cells evaluated by MTT assay. (**F**) Hemolysis rates of PNA-TNG and TPZ@PNA-TNG. (**G**) Body weight changes in mice in each treatment group throughout the experimental period. All data are presented as mean ± SD (n = 3 for in vitro assays; n = 5 per group for in vivo studies).

Safety evaluation of the different interventions was performed through histopathological examination and serum biochemical tests. No appreciable organ damage or toxicity was observed in the TPZ@PNA-TNG group or the other treatment cohorts at the pathological level, relative to the NS control group (Figure 5A). Prior to euthanasia, blood samples were collected from the postocular venous plexus of mice for the measurement of ALT, AST, Cr, and BUN levels. To evaluate potential hepatotoxicity and nephrotoxicity, the measured biochemical parameters were compared with those of the control group. No marked differences in liver or kidney function were observed between the treatment and control groups (*p* > 0.05), indicating that the treatment caused no discernible damage to major organs and demonstrates acceptable biosafety (Figure 5B–D).

The hemolytic activity of PNA-TNG and TPZ@PNA-TNG was evaluated at concentrations of 0.2 mg/mL and 0.0125 mg/mL, respectively. Both formulations induced hemolysis rates below 4% (Figure 5E). In parallel, the cytotoxicity of the nanogels was assessed against 4T1 cells over a concentration range of 3.125–100 μg/mL following 24 h of incubation. Results indicated that the PNA-TNG group maintained a cell survival rate exceeding 80%, while the TPZ@PNA-TNG group exhibited a survival rate below 20%, demonstrating significant inhibitory effects of TPZ@PNA-TNG on 4T1 cells (Figure 5F). Mice were weighed every two days during the observation period. During the experimental period, weight loss was observed in the NS, PNA-TNG, and TPZ groups, presumably resulting from the elevated metabolic requirements of aggressively proliferating tumor cells and their competition with normal cells for nutrients. In contrast, the TPZ@PNA-TNG group maintained nearly unchanged body weights (Figure 5G). The results demonstrate that TPZ@PNA-TNG exhibits excellent biocompatibility.

## 3. Conclusions

In this study, we successfully developed a thermosensitive nanogel system, TPZ@PNA-TNG, for localized breast cancer therapy. The nanogel exhibits a well-defined core–shell nanostructure, favorable temperature-responsive sol–gel transition behavior, and pH-triggered sustained drug release under acidic tumor conditions. Following intratumoral injection, its injectability and in situ gelation behavior allow for durable drug retention and continuous local drug supplementation, thereby enhancing intratumoral drug concentrations while minimizing systemic exposure. In the 4T1 tumor model, TPZ@PNA-TNG effectively suppressed tumor proliferation and angiogenesis, induced extensive necrosis and apoptosis, and inhibited tumor progression. Immunohistochemical and cytokine analyses further indicated that the nanogel was associated with increased T-cell infiltration and elevated levels of pro-inflammatory and antitumor cytokines, implying a potential modulatory effect on the tumor immune microenvironment. In vivo safety assessments confirmed its favorable biocompatibility and low systemic toxicity, supporting its potential for local cancer intervention.

However, several limitations should be noted. First, this study was designed as a short-term local efficacy evaluation in a subcutaneous tumor model; therefore, long-term outcomes such as survival and metastasis were not evaluated. Second, although the observed antitumor effects are consistent with the known hypoxia-activated mechanism of TPZ, direct evidence of tumor hypoxia, TPZ activation, or hypoxia-selective cytotoxicity was not obtained. Third, direct pharmacokinetic data and in vivo retention imaging were not acquired, and intratumoral injection is inherently limited to accessible solid tumors, with potential challenges in drug distribution within large or heterogeneous lesions. Future studies will employ flow cytometry and Western blotting to clarify the molecular pathways underlying the observed immune modulation. Additional in vivo experiments, including long-term therapeutic monitoring, survival analysis, tumor recurrence evaluation, and tissue biodistribution, will be conducted to assess therapeutic durability and pharmacokinetic behavior. Moreover, comprehensive biosafety evaluations encompassing chronic toxicity, immunogenicity, and organ accumulation will be performed to further validate the translational potential of TPZ@PNA-TNG. These follow-up investigations will strengthen the theoretical and experimental foundation for the eventual clinical translation of this thermosensitive nanogel platform.

## 4. Materials and Methods

### 4.1. Materials and Animals

TPZ, NIPAM, MBA, AA, STAC, AAPH and MTT were purchased from Aladdin (Shanghai, China). The constant-temperature-heating magnetic stirrer DF-101S was purchased from Yuhua Instrument Co., Ltd. The freeze dryer FD-1A-50 was purchased from Shanghai Minquan Instrument Co., Ltd. (Gongyi, Henan, China). The NanoZS90 DLS particle size analyzer and the Kinexus Ultra-advanced rotational rheometer were purchased from Malvern Instruments Ltd. (Worcestershire, UK). The UV-Vis spectrophotometer A360 was purchased from Aoyi Instruments (Shanghai) Co., Ltd. (Shanghai, China). TEM Tecnai G2 20 was purchased from FEI (Hillsboro, OR, USA) in the United States. 4T1 (mouse breast cancer cells, BNCC273810, equivalent to ATCC CRL-2539, RRID: CVCL_0125) and RPMI-1640 complete medium were purchased from Pronosa Life Science and Technology Co., Ltd. (Wuhan, China). TNF-α, IFN-γ, IL-6, IL-1β, and HMGB1 ELISA kits were purchased from Lianzu Biotechnology Co., Ltd. (Shanghai, China). PBS buffer and 4% PFA were obtained from Biosharp (Hefei, Anhui, China). Trypsin-EDTA was purchased from Gibco (Grand Island, NY, USA).

BALB/c mice used in this study were purchased from the Laboratory Animal Center of Hubei University of Science and Technology. All animals were acclimatized under standard laboratory conditions (22 ± 2 °C) for at least 7 days prior to the commencement of experiments. All animal experiments were carried out in accordance with the guidelines for the care and use of laboratory animals formulated by the Hubei Provincial Department of Science and Technology. This study was approved by the Animal Ethics Committee of Hubei University of Science and Technology (Grant No. 2024-02-002).

### 4.2. Preparation of the PNA-TNG

NIPAM (1.7 g, 0.015 mol), MBA (0.03 g, 0.195 mmol), STAC (0.05 g, 0.144 mmol), and AAPH (0.08 g, 0.295 mmol) were dissolved in 150 mL of ultrapure water in a three-necked flask. The mixture was stirred at 300 rpm in an oil bath until all reagents were completely dissolved, and then heated to 80 °C. Upon the appearance of a blue coloration, AA (230 μL) was introduced, and polymerization was allowed to proceed for approximately 4 h. The resulting product was dialyzed against ultrapure water for 3 days, transferred to a glass Petri dish, and lyophilized using a vacuum freeze dryer to obtain PNA-TNG.

### 4.3. Structural Morphology and Temperature Sensitivity Characterization of the PNA-TNG

A drop of 5 mg/mL PNA-TNG dispersion was placed onto a copper grid, followed by the addition of 0.1 mg/mL phosphotungstic acid solution for negative staining. After staining for 5 min, the excess liquid was carefully removed with filter paper. The grid was then dried overnight at room temperature and observed under a TEM at an accelerating voltage of 200 kV to evaluate the morphology of PNA-TNG adsorbed on the copper mesh.

For particle size, PDI, and zeta potential measurements, the PNA-TNG aqueous dispersion was diluted to 0.2 wt%. Then, 2 mL of the diluted sample was transferred into a cuvette and measured using DLS, equipped with a He-Ne laser (633 nm) and a fixed 90° detection angle. Prior to measurement, each sample was equilibrated at 25 °C and 37 °C for 120 s to reach thermal equilibrium. The hydrodynamic diameter, PDI, and zeta potential were recorded as a function of temperature. Each measurement was performed in triplicate.

For FTIR analysis, the PNA-TNG was thoroughly ground with KBr in a mortar and then compressed into a transparent pellet under a pressure of 8–10 MPa using a tablet press. Prior to spectral acquisition, the Fourier transform infrared spectrometer was preheated for 30 min to ensure instrumental stability, and a background spectrum was recorded using a pure KBr pellet as the reference. Sample spectra were collected over the wavenumber range of 400–4000 cm^−1^ at a resolution of 4 cm^−1^, with 4 scans co-added per sample, followed by baseline correction. The characteristic absorption bands were subsequently assigned to their corresponding functional groups, thereby confirming the chemical structure of PNA-TNG.

Rheological measurements were performed using a rheometer equipped with a parallel plate geometry (PP50, diameter: 50 mm, gap: 0.5 mm). Conventional shear viscosity measurements were conducted under the following conditions: temperature: 25 °C, equilibration time: 5 min, shear rate: 300 s^−1^, and measurement duration: 2 min. Shear rate sweep measurements were performed under the following conditions: temperature: 25 °C, equilibration time: 2 min, and shear rate range: 0–588 s^−1^. A total of 31 data points were collected. Dynamic temperature ramp measurements were carried out under the following conditions: equilibration time: 5 min, temperature range: 25–45 °C, frequency: 1 Hz, shear stress: 0.5 Pa, and heating rate: 2 °C/min. To characterize the temperature sensitivity and sol–gel transition behavior of PNA-TNG, the phase transition process was visually monitored and recorded at 25, 30, 31, 32, 33, 34, 35, 36, 37, and 40 °C. In addition, the temperature-induced gelation of the PNA-TNG dispersion at 25 °C and 37 °C was documented using a syringe connected to a catheter.

### 4.4. Screening of TPZ Loading Concentration and Preparation of the TPZ@PNA-TNG

An appropriate amount of PNA-TNG lyophilized powder was accurately weighed and dissolved in ultrapure water to prepare a 6 wt% gel dispersion. A series of TPZ@PNA-TNG samples with TPZ concentrations of 1, 3, 5, and 7 mg/mL were prepared under dark conditions with stirring. Each sample was transferred into glass vials and thermally equilibrated at 25, 32, 34, 37, and 40 °C in sequence. Macroscopic images were recorded at each temperature to document the sol–gel transition behavior of formulations with different drug loadings upon heating.

Meanwhile, the four groups of samples were stored statically at room temperature in the dark, and their macroscopic appearance was examined on days 1, 3, 7, and 10 to observe possible stratification, allowing for the assessment of long-term storage stability. Furthermore, TPZ@PNA-TNG dispersions with TPZ concentrations of 1, 3, and 5 mg/mL were dropped onto clean glass slides and observed under an optical microscope at magnifications of ×100 and ×200. The droplet size, distribution density, and microscopic morphology were comparatively analyzed.

The screening results indicated that low-concentration groups exhibited insignificant phase transition behavior, while high-concentration groups showed poor dispersion uniformity and obvious stratification during storage. In contrast, the 3 mg/mL group displayed uniform microscopic morphology, stable dispersion, and desirable temperature-responsive sol–gel transition performance. Based on a comprehensive evaluation of sol–gel transition behavior, storage stability, and microscopic morphology, 3 mg/mL was selected as the optimal TPZ loading concentration. Using the optimized formulation, TPZ powder was added to the 6 wt% PNA-TNG dispersion to achieve a final TPZ concentration of 3 mg/mL. The mixture was stirred at 250 rpm under dark conditions to obtain the final TPZ@PNA-TNG.

To determine the DL% and EE% of TPZ@PNA-TNG, a TPZ solution (3 mg/mL) was mixed with a 6 wt% PNA-TNG dispersion and stirred at room temperature in the dark for 24 h to achieve complete drug loading. To remove unencapsulated and surface-adsorbed free TPZ, the resulting dispersion was transferred into a dialysis bag (MWCO 12–14 kDa) and dialyzed against deionized water for 4 h. After dialysis, the sample was pre-frozen at −4 °C for 12 h and then lyophilized for 24 h. The obtained lyophilized powder was redispersed in PBS (pH 7.4), and the TPZ concentration was quantified by UV-Vis spectrophotometry at 266 nm. EE% and DL% were calculated using the formulas presented below. All measurements were performed in triplicate.

(1) $$EE\left(\%\right)=\frac{W_{i}-W_{f}}{W_{i}}\times 100\%$$

(2) $$DL\left(\%\right)=\frac{W_{enc}}{W_{NG}{+W}_{enc}}\times 100\%$$

where *W*i is the total mass of TPZ initially added during the drug loading step; *W*_f_ is the mass of unentrapped TPZ quantified in the dialysate after dialysis purification; *W*_enc_ is the mass of TPZ entrapped in the lyophilized TPZ@PNA-TNG determined by UV-Vis spectrophotometry; and *W*_NG_ is the dry mass of the blank PNA-TNG nanogel carrier. To evaluate batch-to-batch reproducibility, three independent batches of TPZ@PNA-TNG were prepared under identical conditions, and the particle size, PDI, zeta potential, EE%, and DL% were determined for each batch.

### 4.5. Characterization and Temperature Sensitivity of TPZ@PNA-TNG Formulations

Temperature-ramp rheological scanning experiments of TPZ@PNA-TNG were performed on a rotational rheometer equipped with a PP50 parallel-plate fixture (plate diameter: 50 mm, gap height: 0.5 mm). After thermal equilibration for 5 min at the initial temperature, the sample was heated from 25 °C to 50 °C at a heating rate of 2 °C/min under a fixed oscillation frequency of 1 Hz and a shear stress of 0.5 Pa. The storage modulus (G′) and loss modulus (G″) were recorded in real time throughout the test to determine the sol–gel transition temperature. Meanwhile, macroscopic photographs of TPZ@PNA-TNG hydrogel at 25 °C and 37 °C were captured for morphological observation. UV-Vis spectrophotometry was used to acquire the absorption spectra of free TPZ solution, PNA-TNG dispersion, and TPZ@PNA-TNG suspension, respectively.

For particle size and Zeta potential measurements, TPZ@PNA-TNG was diluted with ultrapure water to 0.2 wt%. A 2 mL aliquot of the diluted sample was loaded into the dedicated sample cell of a laser particle size analyzer equipped with a He-Ne laser and operated at a 90° detection angle. The particle size distribution curves were measured at 25 °C and 37 °C, respectively. Meanwhile, the zeta potential distribution was determined under these two temperature conditions. For transmittance measurements, TPZ@PNA-TNG (50 mg) was added to 10 mL of ultrapure water and fully dispersed by stirring under dark conditions to prepare a 5 mg/mL aqueous dispersion. A 2 mL aliquot of the dispersion was transferred into a quartz cuvette. The sample temperature was precisely regulated with a thermostatic water bath, and the transmittance was recorded using a UV-Vis spectrophotometer. Macroscopic photographs of the sample within the cuvette were also acquired at 25 °C, 34 °C, and 37 °C.

### 4.6. Drug Release

Subsequently, 2 mL of the 6wt% TPZ@PNA-TNG aqueous gel dispersion was transferred into release media at pH 5.8, 7.4, and 8.8, respectively. The samples were incubated in a constant-temperature shaker at 37 °C with continuous agitation at a fixed speed. At predetermined time intervals (1, 2, 3, 6, 12, 24, 48, 72, 96, and 120 h), 3 mL aliquots were withdrawn from each release medium, and an equal volume of fresh medium of the corresponding pH was immediately replenished. The collected samples were analyzed via UV-Vis spectrophotometry at 270 nm. The concentration of TPZ@PNA-TNG in each withdrawn sample was determined from the absorbance measurements, and the cumulative release rate was calculated as follows:

(3) $$Cumulative\;drug\;release\;rate\left(\%\right)=\frac{Q_{t}}{Q_{total}}\times 100\%$$

where *Q*_t_ is the cumulative amount of TPZ released at time *t*, and *Q*_total_ is the total amount of TPZ initially loaded in TPZ@PNA-TNG.

### 4.7. Mice with 4T1 Subcutaneous Tumor Were Modeled and Administered by Group

To establish the 4T1 subcutaneous tumor model, 4T1 cells in the logarithmic growth phase were digested with trypsin until they became rounded and detached, and the digestion was terminated by the addition of RPMI-1640 complete medium. The cell suspension was centrifuged at 1000 rpm for 3 min, and the supernatant was discarded. The cells were then resuspended in PBS to obtain a concentration of 1 × 10^6^ cells/mL. Subsequently, 0.1 mL of the cell suspension was slowly injected into the dorsal thigh region of BALB/c mice. Tumor volume was measured daily and calculated using the following formula:

(4) V=π/6×L×W×H

where *L*, *W*, and *H* represent the length, width, and height of the tumor, respectively. When the tumor volume reached 150–200 mm^3^, the mice were randomly divided into four groups (n = 5 per group): NS, PNA-TNG, TPZ, and TPZ@PNA-TNG. From day 1 after grouping, TPZ or TPZ@PNA-TNG was administered via intratumoral injection at a TPZ dose of 10 mg/kg in a volume of 100 μL per mouse, every other day on days 1, 3, 5, and 7 (four doses in total). The NS and PNA-TNG groups received equivalent volumes of saline or blank nanogel. Tumor volumes and body weights were measured daily throughout the 14-day experimental period. On day 14, all mice were euthanized, and tumor tissues were collected for further analysis.

### 4.8. Evaluation of Immunofluorescence and Histopathology

After 14 days of treatment, all mice were euthanized, and tumor tissues were carefully excised under sterile conditions. Tumor tissue samples were collected from the central region of the tumor mass and immediately fixed in 4% PFA at 4 °C for 12–24 h to preserve tissue morphology. Immunohistochemical staining was performed to evaluate TUNEL, Ki67, VEGF, CD31, CD3, CD4, CRT, and CD8, and the average fluorescence intensity was quantified for each marker. Additionally, serum samples were collected for cytokine analysis. The levels of TNF-α, HMGB1, IFN-γ, IL-1β, and IL-6 were determined using ELISA kits, with absorbance measured at 450 nm on a microplate reader.

### 4.9. Liver and Kidney Function Tests

Blood samples collected from the postocular venous plexus were allowed to clot at room temperature for 30 min and then centrifuged at 3000 rpm for 10 min. The resulting serum was transferred to fresh microcentrifuge tubes. The activities of ALT and AST were determined using an automated biochemical analyzer, following the manufacturer’s protocols. Serum levels of BUN and Cr were also measured to evaluate renal function.

### 4.10. Biocompatibility Analysis

4T1 cells in the logarithmic growth phase of 4T1 cells was digested with trypsin and resuspended in complete medium to achieve a cell concentration of 5 × 10^3^ cells/mL. A 100 μL aliquot of the cell suspension was added per well in 96-well plates, resulting in approximately 500–1000 cells per well. The plates were incubated at 37 °C for 24 h to allow cell adhesion. After 24 h, the original medium was discarded, and 100 μL of TPZ@PNA-TNG or PNA-TNG nanogel solutions at various concentrations was added per well. A control group receiving an equal volume of complete medium was also established. Three replicate wells were set for each concentration, and the plates were incubated for another 24 h. The negative control consisted of untreated 4T1 cells, and the blank control consisted of medium without cells. Four hours before the end of incubation, 20 μL of MTT solution was added to each well, followed by an additional 4 h of incubation. After incubation, the medium was discarded, and 150 μL of DMSO was added to each well. The plates were shaken at room temperature for 10 min, and the absorbance was then recorded at 570 nm using a microplate reader. The absorbance values of the experimental groups (PNA-TNG and TPZ@PNA-TNG), blank control (medium only), and negative control (untreated cells) were denoted as *T*, *B*, and *C*, respectively. Cell viability was calculated using the following formula:

(5) $$\mathrm{Cell}\;\mathrm{survival}\;\mathrm{rate}\;\left(\%\right)=\frac{T-B}{C-B}\times 100\%$$

A 2% RBC suspension was prepared by mixing 2 mL of rabbit whole blood with NS and centrifuging at 1500 rpm for 25 min. The TPZ@PNA-TNG and PNA-TNG nanogel solutions were diluted with NS to obtain a series of sample solutions at different concentrations for the experimental groups. NS and ultrapure water were used as the negative and positive controls, respectively. Subsequently, 150 µL of each sample solution and 150 µL of the 2% RBC suspension were added to centrifuge tubes. The mixtures were incubated in a water bath at 37 °C and then centrifuged at 3000 rpm for 25 min to collect the supernatant. The absorbance of the supernatant was measured at 540 nm using an EIA plate reader (ELX800, Bio-Tek Instruments, Winooski, VT, USA). The hemolysis rate was calculated as follows:

(6) $$\mathrm{Hemolysis}\;\mathrm{rate}\;\left(\%\right)=\frac{A_{s}-A_{n}}{A_{p}-A_{n}}\times 100\%$$

where *A*_s_, *A*_n_, and *A*_p_ are the absorbances of the sample, negative control (NS), and positive control (ultrapure water), respectively.

### 4.11. Statistical Analysis

All data are presented as the mean ± SD. Statistical analyses were performed using SPSS (version 28.0) and GraphPad Prism (version 6.01). For comparisons among multiple groups, one-way ANOVA was performed, followed by Dunnett’s post-hoc test for pairwise comparisons between each group (NS, PNA-TNG, and TPZ) and the TPZ@PNA-TNG group. Average fluorescence intensity was quantified using ImageJ 1.54f (National Institutes of Health, USA). Significance levels are indicated as follows: *: *p* < 0.05, **: *p* < 0.01, ***: *p* < 0.001, and ****: *p* < 0.0001.

## Data Availability

The data presented in this study are available on request from the corresponding authors. The data are not publicly available.

## Supplementary Materials

The following supporting information can be downloaded at https://www.mdpi.com/article/10.3390/gels12090781/s1, Figure S1: Phase transition diagram of the PNA-TNG; Figure S2: Thermoresponsive Behavior of PNA Nanogels; Vidio S1: The temperature sensitivity of the PNA-TNG at 25 °C; Video S2: The temperature sensitivity of the PNA-TNG at 37 °C; Figure S3: Phase change diagram of % TPZ@PNA-TNG emulsion; Figure S4: In vitro stability of TPZ@PNA-TNG; Figure S5: Optical microscopic morphology of TPZ@PNA‑TNG prepared at different TPZ feeding concentrations (1 mg/mL, 3 mg/mL, and 5 mg/mL). Upper panels: ×100 magnification, scale bar = 100 μm; lower panels: ×200 magnification, scale bar = 50 μm; Figure S6: The encapsulation efficiency (EE) and drug-loading content (DL) of TPZ@PNA-TNG nanogel. Values represent mean±standard deviation (n = 3); Figure S7: Immunofluorescence staining for CD3 (left) and quantitative fluorescence intensity across treatment groups (right); Figure S8: Immunofluorescence staining for CD8 (left) and quantitative fluorescence intensity across treatment groups (right); Figure S9: Immunofluorescence staining for CRT (left) and quantitative fluorescence intensity across treatment groups (right); Figure S10: Immunofluorescence staining for IL-1β (left) and quantitative fluorescence intensity across treatment groups (right); Figure S11: Immunofluorescence staining for CD4 (left) and quantitative fluorescence intensity across treatment groups (right); Figure S12: Immunofluorescence staining for IFN-γ (left) and quantitative fluorescence intensity across treatment groups (right); Figure S13: Immunofluorescence staining for HMGB-1 (left) and quantitative fluorescence intensity across treatment groups (right).

## Abbreviations

4T1, 4T1 murine breast cancer cell; AA, acrylic acid; AAPH, 2,2′-azobis(2-methylpropamidine) dihydrochloride; ALT, alanine aminotransferase; ANOVA, analysis of variance; AST, aspartate aminotransferase; BUN, blood urea nitrogen; Cr, creatinine; CRT, calreticulin; DC, dendritic cell; DL, drug loading; DLS, dynamic light scattering; DMSO, dimethyl sulfoxide; EE, encapsulation efficiency; EDTA, ethylenediaminetetraacetic acid; EIA, enzyme immunoassay; ELISA, enzyme-linked immunosorbent assay; FTIR, Fourier transform infrared spectroscopy; H&E, hematoxylin and eosin; HMGB1, high mobility group box 1; ICD, immunogenic cell death; IFN-γ, interferon-γ; IL-1β, interleukin-1β; IL-6, interleukin-6; KBr, potassium bromide; MBA, N,N’-methylenebisacrylamide; MTT, 3-(4,5-dimethylthiazol-2-yl)-2,5-diphenyltetrazolium bromide; NIPAM, N-isopropylacrylamide; NK, natural killer; NS, normal saline; PBS, phosphate-buffered saline; PDI, polydispersity index; PFA, paraformaldehyde; PNA-TNG, poly(N-isopropylacrylamide)-co-acrylic acid thermosensitive nanogel; RBC, red blood cell; SD, standard deviation; SPSS, Statistical Product and Service Solutions; STAC, octadecyl trimethyl ammonium chloride; TACE, transarterial chemoembolization; TEM, transmission electron microscopy; TNF-α, tumor necrosis factor-α; TPZ, tirapazamine; TPZ@PNA-TNG, TPZ loaded PNA-TNG nanogel; TUNEL, terminal deoxynucleotidyl transferase dUTP nick end labeling; UV-Vis, ultraviolet-visible; VEGF, vascular endothelial growth factor.
