# Supplementary material for: In Situ Gelling of TPZ-Loaded Nanogel for Sustained Intratumoral Delivery and Enhanced Cancer Immunotherapy
Source: Gels. 2026 Sep 1;12(9):781. doi: 10.3390/gels12090781 (PMC13606876; doi:10.3390/gels12090781)
Supplement: Supplementary file 1 [file gels-12-00781-s001.zip › gels-4464047-supplementary.pdf]

Supplementary Files

In Situ Gelling TPZ-Loaded Nanogel for Sustained Intratumoral Delivery and Enhanced Cancer Immunotherapy

Ling Li [1,†], Kexin Wang [1,2,†], Zhe Song [3], Hongan Tian [3], Cai Wang [1], Ling Zhang [1] and Houqiang Yu [1,4,*]

[1] School of Biomedical Engineering, Xianning Medical College, Hubei University of Science and Technology, Xianning 437100, China

[2] School of Pharmacy, Xianning Medical College, Hubei University of Science and Technology, Xianning, Hubei, 437100, China

[3] Xianning Medical College, Hubei University of Science and Technology, Xianning 437100, China

[4] Department of Mathematics and Statistics, Hubei University of Science and Technology, Xianning 437100, China

* Correspondence: yuhouqiang@hbust.edu.cn

† These authors contributed equally to this work.

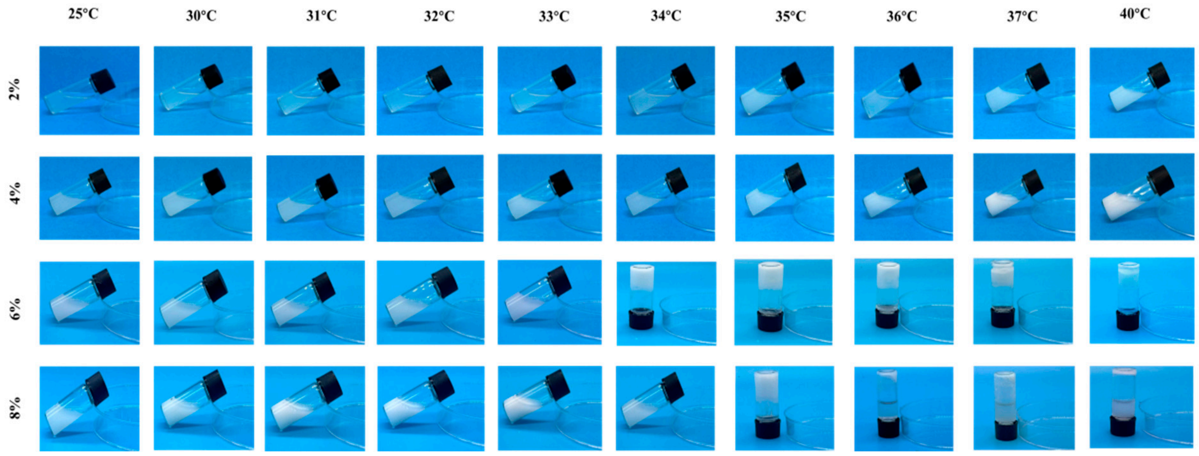

Figure S1. Phase transition diagram of the PNA-TNG

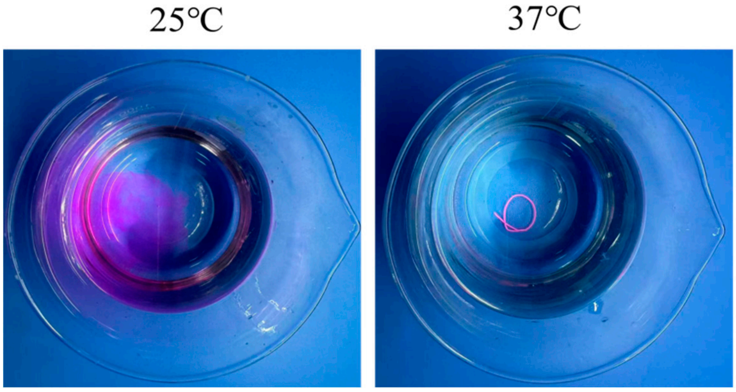

Figure S2. Thermoresponsive Behavior of PNA Nanogels

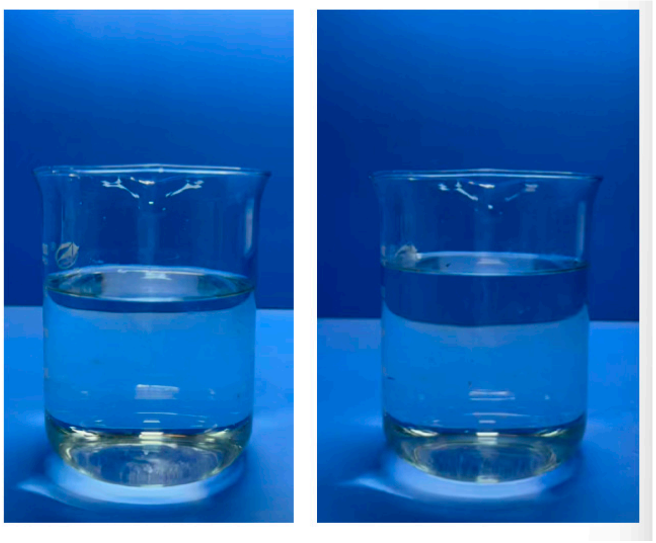

Video S1. The temperature sensitivity of the PNA-TNG at 25°C

Video S2. The temperature sensitivity of the PNA-TNG at 37°C

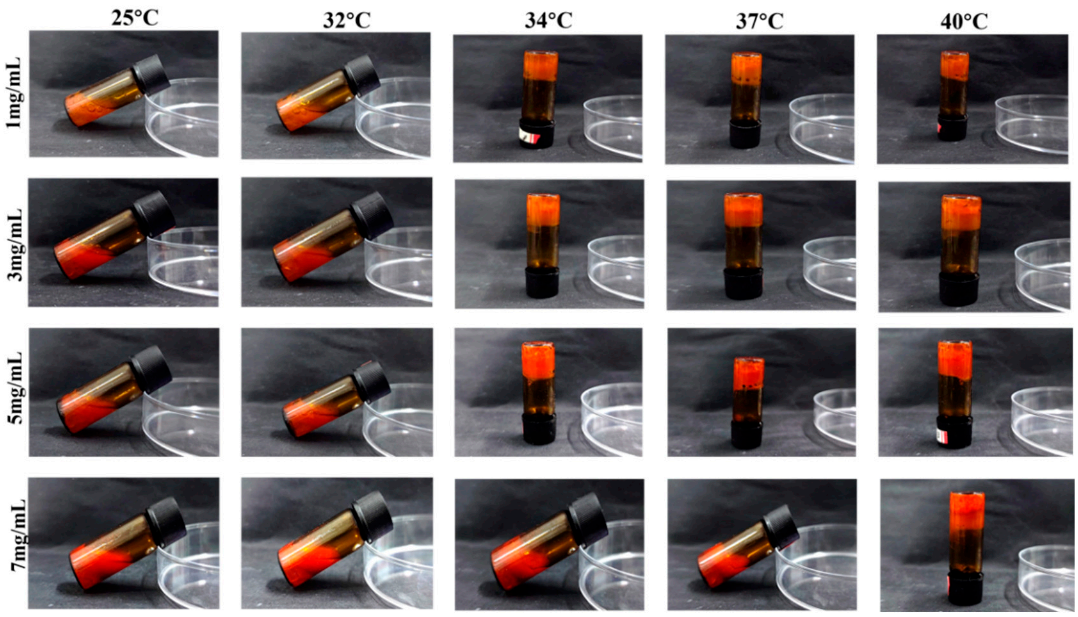

Figure S3. Phase change diagram of % TPZ@PNA-TNG emulsion

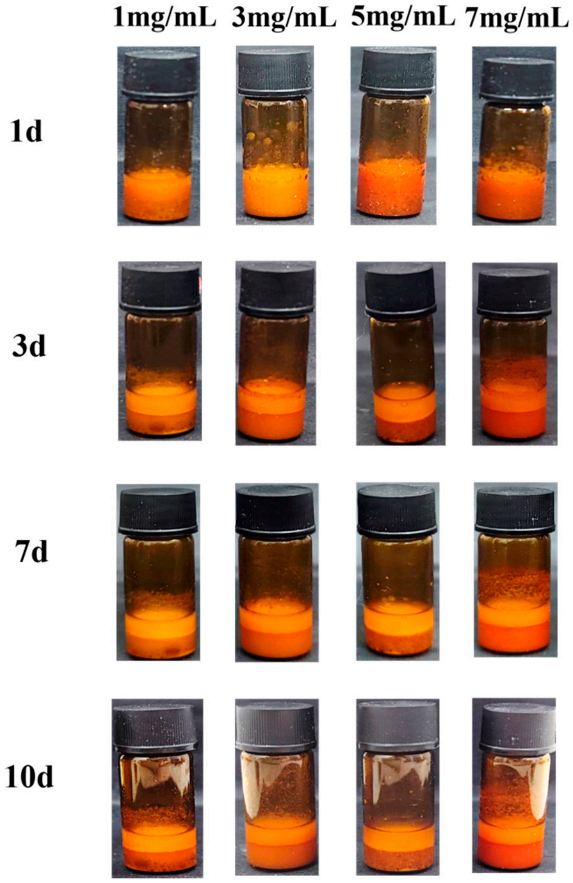

Figure S4. In vitro stability of TPZ@PNA-TNG

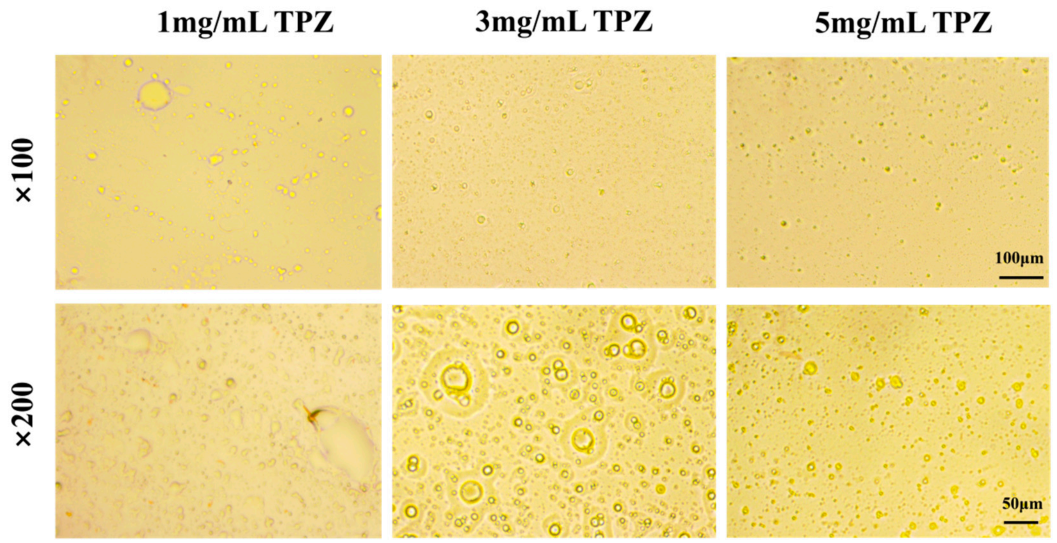

Figure S5. Optical microscopic morphology of TPZ@PNA-TNG prepared at different TPZ feeding concentrations (1 mg/mL, 3 mg/mL, and 5 mg/mL). Upper panels: ×100 magnification, scale bar = 100 μm; lower panels: ×200 magnification, scale bar = 50 μm.

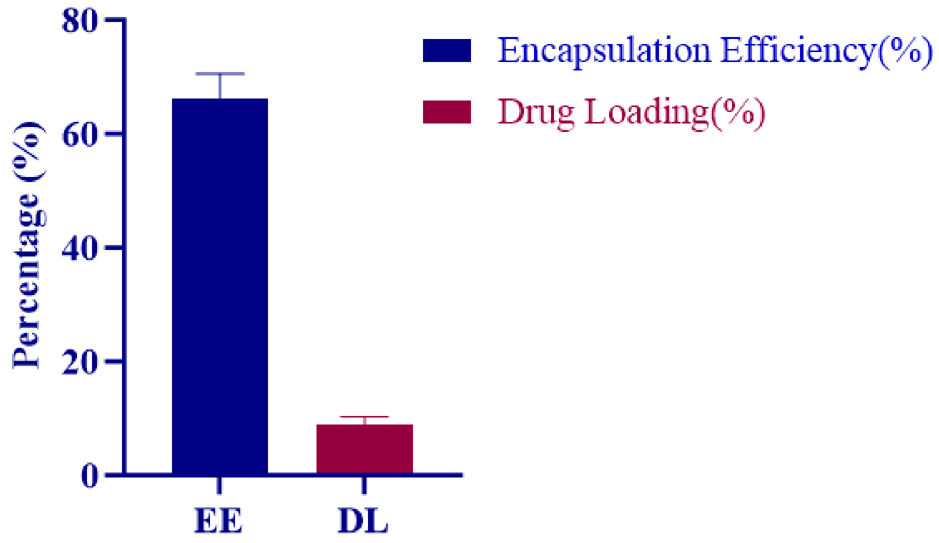

Figure S6. The encapsulation efficiency (EE) and drug-loading content (DL) of TPZ@PNA-TNG nanogel. Values represent mean ± standard deviation (n = 3).

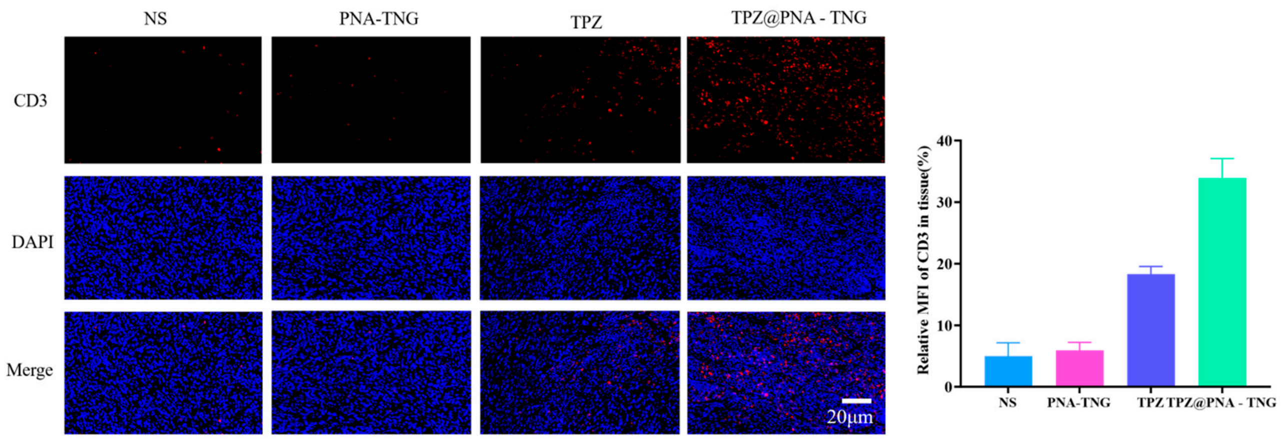

Figure S7. Immunofluorescence staining for CD3 (left) and quantitative fluorescence intensity across treatment groups (right).

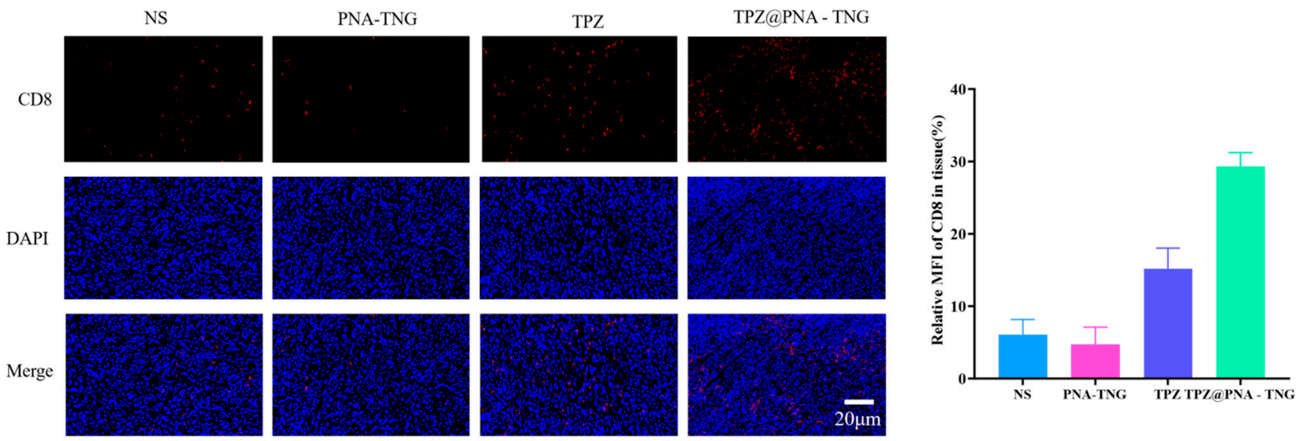

Figure S8. Immunofluorescence staining for CD8 (left) and quantitative fluorescence intensity across treatment groups (right).

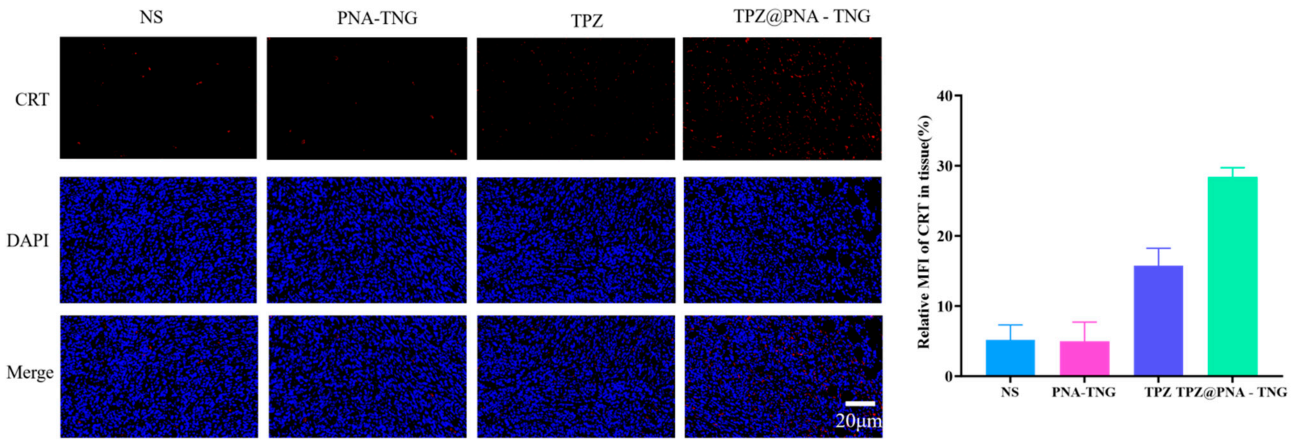

Figure S9. Immunofluorescence staining for CRT (left) and quantitative fluorescence intensity across treatment groups (right).

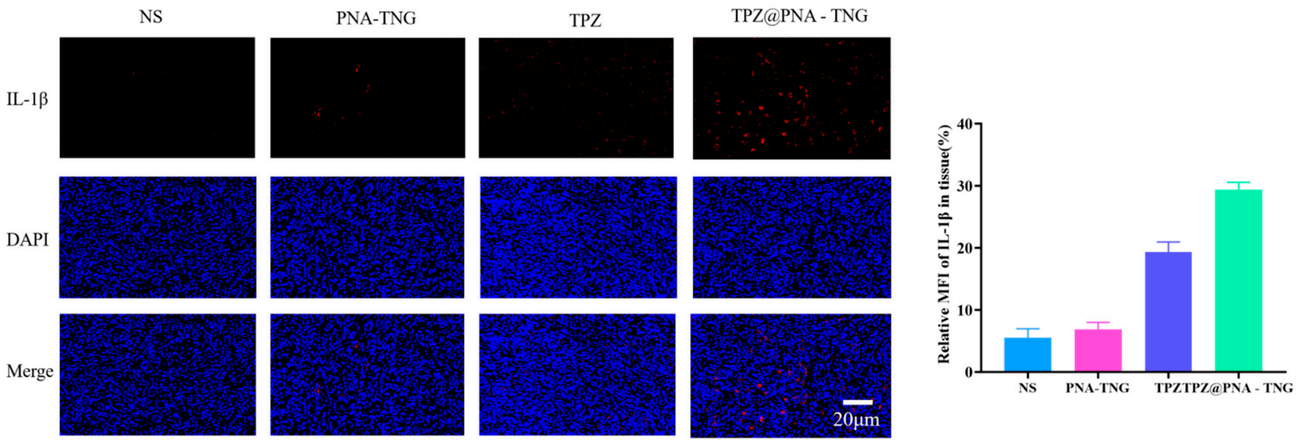

Figure S10. Immunofluorescence staining for IL-1β (left) and quantitative fluorescence intensity across treatment groups (right).

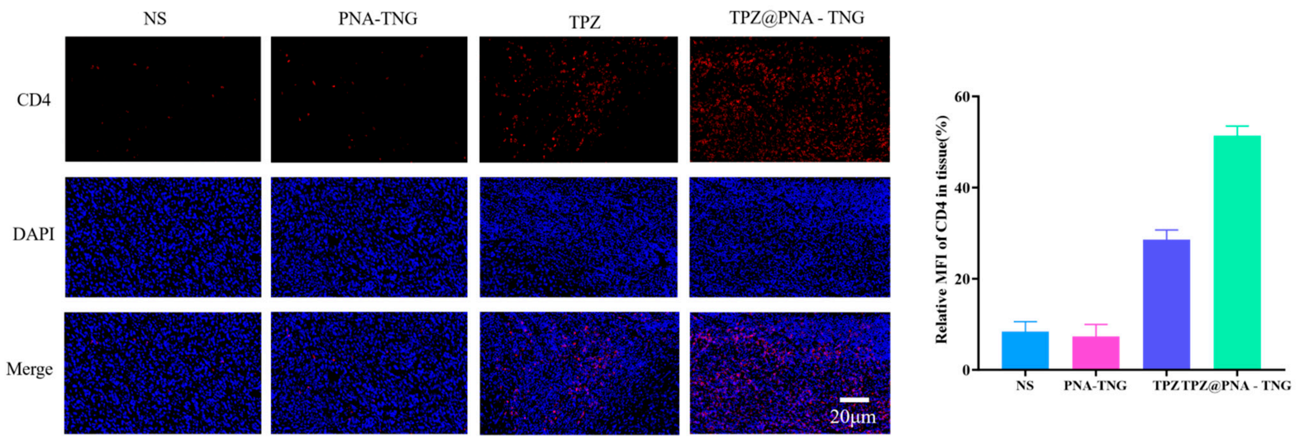

Figure S11. Immunofluorescence staining for CD4 (left) and quantitative fluorescence intensity across treatment groups (right).

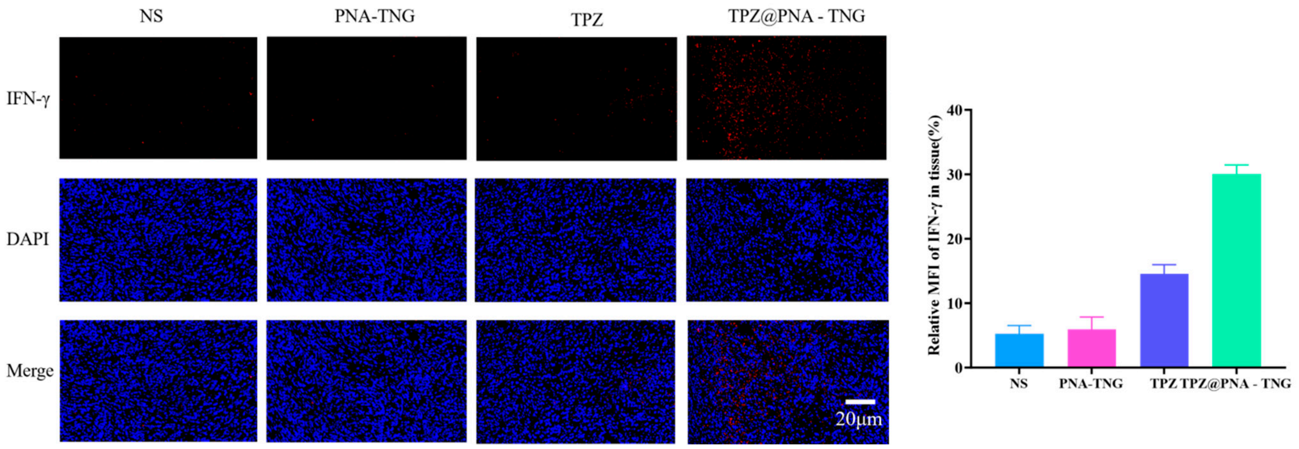

Figure S12. Immunofluorescence staining for IFN-γ (left) and quantitative fluorescence intensity across treatment groups (right).

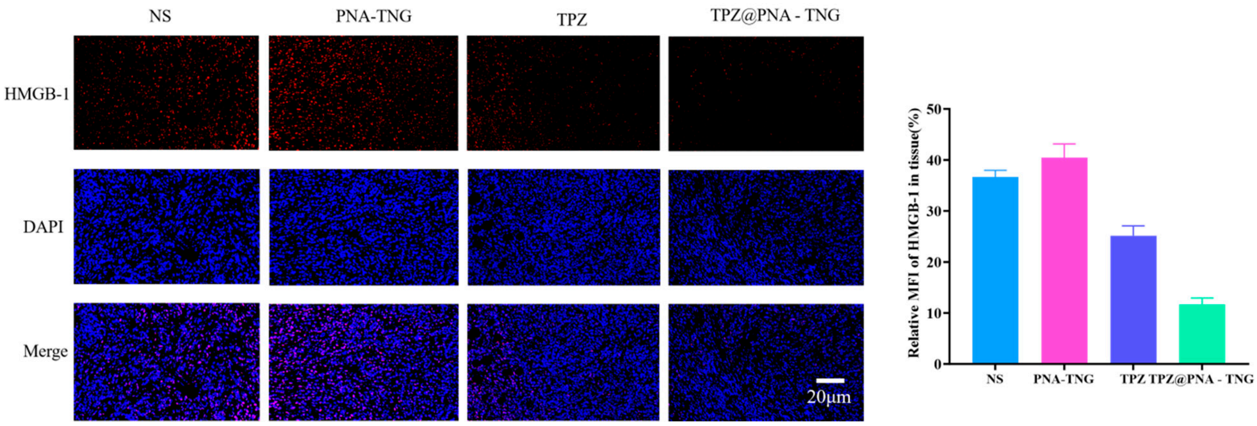

Figure S13. Immunofluorescence staining for HMGB-1 (left) and quantitative fluorescence intensity across treatment groups (right).
